# Multi-Parametric Relationships between PAM Measurements and Carbon Incorporation, an *In Situ* Approach

**DOI:** 10.1371/journal.pone.0040284

**Published:** 2012-07-20

**Authors:** Camille Napoléon, Pascal Claquin

**Affiliations:** 1 Université de Caen Basse-Normandie, BIOMEA FRE3484 CNRS, Caen, France; 2 CNRS INEE, FRE3484 BIOMEA, Caen, France; 3 IFREMER, Laboratoire Environnement Ressources de Normandie, Avenue du Général de Gaulle, Port-en-Bessin, France; US Dept. of Agriculture – Agricultural Research Service (USDA-ARS), United States of America

## Abstract

Primary production (PP) in the English Channel was measured using ^13^C uptake and compared to the electron transport rate (ETR) measured using PAM (pulse amplitude modulated fluorometer). The relationship between carbon incorporation (P_obs_) and ETR was not linear but logarithmic. This result can be explained by alternative electron sinks at high irradiance which protect the phytoplankton from photoinhibition. A multi-parametric model was developed to estimate PP by ETR. This approach highlighted the importance of taking physicochemical parameters like incident light and nutrient concentrations into account. The variation in the ETR/P_obs_ ratio as a function of the light revealed different trends which were characterized by three parameters (R_max_, the maximum value of ETR/P_obs_; E_Rmax_, the light intensity at which R_max_ is measured; γ the initial slope of the curve). Based on the values of these three parameters, data were divided into six groups which were highly dependent on the seasons and on the physicochemical conditions. Using the multi-parametric model which we defined by P_obs_ and ETR measurements at low frequencies, the high frequency measurements of ETR enabled us to estimate the primary production capacity between November 2009 and December 2010 at high temporal and spatial scales.

## Introduction

Primary production forms the base of the marine food web. Consequently, every trophic level depends on it [1] and a reliable estimation of primary production is indispensable for understanding and creating models of marine ecosystems. However, numerous environmental factors control the dynamics of primary production [2], [3], which makes its estimation difficult.

Remote sensing is commonly used to estimate primary production by using stock data of chlorophyll *a* [4], [5], [6] but results have rarely been validated by *in situ* measurements. Grangeré et al. [7] showed underestimation of primary production by using chlorophyll *a* data. To obtain a precise estimation of primary production, *in situ* measurements are essential. Different methods can be used to make *in situ* measurements of primary production, each of which has advantages and disadvantages. One such method is labelled carbon incorporation [8], [9]. This method is sensitive but cannot be used for measurements at large spatiotemporal scales due to its long incubation period. Yet the study of the spatiotemporal dynamics of primary production requires data at large spatiotemporal scales. The PAM (pulse amplitude modulated fluorometer) method based on the variation in chlorophyll *a* fluorescence in the Photosystem II is more flexible as it allows rapid measurements of photosynthetic parameters and estimates the physiological state of the phytoplankton [10], [11]. This in turn, means that phytoplankton productivity can be monitored at large spatial and temporal scales. In addition, PAM is sensitive and non-invasive.

The labelled carbon incorporation method enables the incorporation of dissolved inorganic carbon into organic matter to be measured whereas the PAM method does not give the rate of photosynthetic carbon incorporation directly [12], [13] but enables access to the electrons transport rate (ETR) from the PSII. Combining these two approaches results in a very powerful tool to estimate carbon assimilation at large spatial and temporal scales. By combining the fluorescence approach and traditional incubation methods, it is possible to estimate the potential production of carbon knowing the electrons flux [12], [14], [15]. But this relation is not trivial. Environmental factors do not all affect ETR in the same way, so carbon fixation and therefore the number of electrons required to fix one mol of carbon is not constant. For example, the maximum quantum yield of carbon fixation varies as a function of the nitrate concentration [16] or temperature [17]. Various physiological processes can hinder the flow of electrons among others, the Mehler reaction, chlororespiration, photorespiration, and nitrate fixation [12], [18], [19], in response to environmental changes or as function of the species composition [20].

Many studies have shown that it is possible to use the fluorescence approach by comparing it to other traditional incubation methods to estimate primary production such as labelled carbon incorporation or oxygen measurements [10], [12], [17], [21], [22]. By contrast, only a few authors have analysed the effect of physicochemical [16] or biological parameters [14] on the conversion of photosynthetic electron transport rates (ETR) into carbon fixation rates.

In the present study, we investigated the ETR and ^13^C incorporation relationships over one year on a transect in the central English Channel [23]. The objectives of the present study were to: i) describe the influence of physicochemical and biological parameters on the relationship between the PAM method and the carbon incubation method, ii) estimate the rate of carbon fixation as a function of ETR using a multi-parametric approach which allows the influence of physicochemical and biological parameters to be taken into account, and to hierarchize them, iii) apply the relationship obtained between carbon fixation rate and ETR on the whole PAM dataset measured at high frequency in the central English Channel between November 2009 and December 2010 [23].

## Materials and Methods

### 1. Study Area

As described in Napoléon et al. [23], measurements were made every month from November 2009 to December 2010 in the central part of the English Channel. The English Channel in North-West Europe is an epicontinental sea which is connected to the North Sea in the north and influenced by the Atlantic Ocean in the west. Napoléon et al. [23] characterised different areas in the central part of the English Channel, revealing that their functioning differed depending on freshwater inputs from rivers or on the influence of offshore water. These different hydrographic conditions drive the dynamics of photosynthetic parameters. Consequently, it is interesting to study primary production in this complex system which comprises both rich and depleted areas.

**Figure 1:**
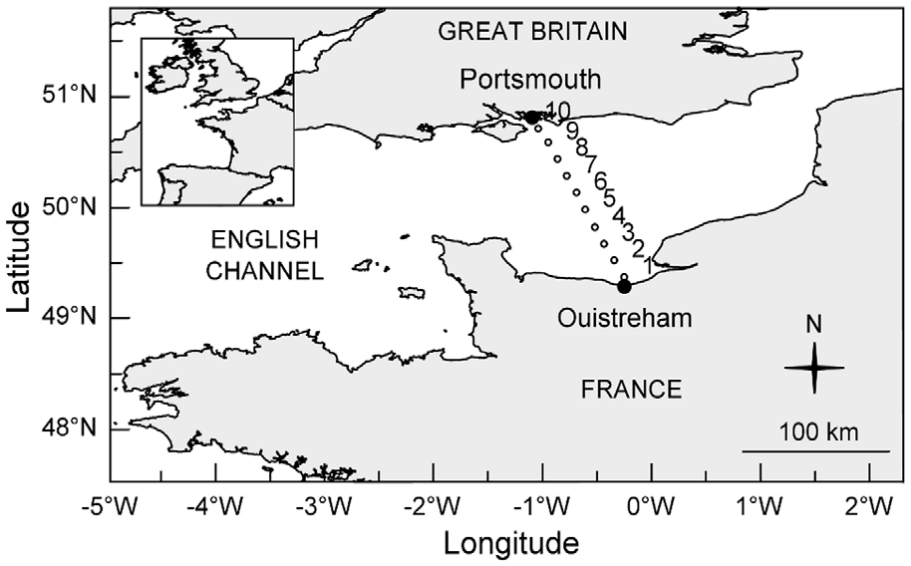
Map of the sampling area.

Data were collected onboard the *Normandie-Brittany ferries* at a depth of four metres during the ferry’s daily cruises on a 175 kilometres transect between Ouistreham (France, 49°17′27 N, 000°14′45 W) and Portsmouth (Great Britain, 50°48′49 N, 001°05′29 W) (Figure 1). Parameters were recorded at the 10 stations shown on the map in Figure 1.

### 2. Physicochemical Parameters

As described in detail in Napoléon et al. [23], temperature and salinity were recorded with an YSI 6600 V2 multi-parameter probe, and light was measured on the deck with a 2π PAR sensor LI-192 connected to a data Logger LI-1400 (LI-COR, Lincoln, Nebraska, USA). Dissolved inorganic nitrogen (DIN), phosphate (DIP) and silicate (DSi) concentrations were determined in the laboratory using a AA3 auto analyser (AXFLOW) following the method of Aminot and Kérouel [24]. Suspended particulate matter (SPM) was measured following the method of Aminot and Chaussepied [25].

### 3. Biological Parameters

The chlorophyll *a* (Chl*a*) concentration was measured according to the method of Welschmeyer [26] as described in Napoléon et al. [23]. Phytoplankton were identified in water samples preserved by Lugol’s solution (2 mL L^−1^) using the Utermöhl method. The dinoflagellate:diatom ratio was calculated for each sample.

To obtain the chlorophyll-specific absorption cross section (a*; m^2^ mg Chl*a* ^−1^), one litre of seawater was filtered onto a GF/F glass-fibre filter. The *in vivo* optical density of total particles (OD_t_) from 400 to 750 nm was measured directly on the filter using a Perkin Elmer integrating sphere spectrophotometer [27]. The sample was recovered with methyl alcohol (MeOH) to extract phytoplankton pigments [28]. After one hour, the *in vivo* optical density for non-algal particles (OD_np_) was measured directly on the filter rinsed with filtered seawater. The optical density of the phytoplankton (OD_p_) was determined using:
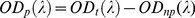
(1)


The chlorophyll-specific absorption cross section (a*; m^2^ mg Chl*a* ^−1^) was calculated using the equation of Johnsen and Sakshaug [29]:
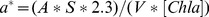
(2)where A is the average OD_p_ between 400 nm and 700 nm, S is the clearance area of the filter (1256 mm^2^), V is the filtered volume in mL and the chlorophyll *a* concentration is in mg m^−3^. a_ph_ in m^−1^ was determined according to [30]:
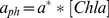
(3)where the chlorophyll *a* concentration is in mg m^−3^.

### 4. PAM Fluorometry

The maximum energy conversion efficiency, or quantum efficiency of PSII charge separation (F_v_/F_m_) was measured using the flow-through (FT) version of WATER PAM (Walz, Effeltrich, Germany) [31]. As described in Napoléon et al. [23], the water collected at a depth of 4 m was conducted through a pipe to a 100 mL dark tank. After 10 min of dark acclimation, a 30 mL sub-sample was automatically transferred into the measuring chamber. The sample was excited by a weak blue light (1 µmol photons m^−2^ s^−1^, 470 nm, frequency 0.6 kHz) to record the minimal fluorescence (F_0_). The maximum fluorescence (F_m_) was obtained during a saturating light pulse (0.6 s, 1700 µmol photons m^−2^ s^−1^, 470 nm), allowing the quinone A (QA) pool to be reduced. F_v_/F_m_ was calculated according to the following equation [32] after subtraction of the blank fluorescence, measured on seawater filtrated through GF/F glass-fibre filter:
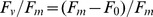
(4)


The samples were exposed to nine irradiances (E) from 0 to 1000 µmol photons m^−2^ s^−1^ for 55 s at each step. Steady state fluorescence (F_s_) and maximum fluorescence (F_m_’) were measured. The effective quantum efficiency of PSII for each irradiance was determined as follows [32]:
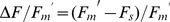
(5)


The relative electron transport rate (rETR, relative unit) was calculated for each irradiance. rETR is a measure of the rate of linear electron transport through Photosystem II, which is correlated with the overall photosynthetic performance of the phytoplankton [33]:
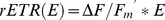
(6)


The electron transport rate (ETR) in µmol electron L^−1^ h^−1^ was calculated as follows:
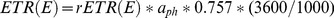
(7)where a_ph_ is in m^−1^. According to Johnsen and Sakshaug [29] and knowing the species composition, which was largely dominated by diatoms and Dinophyta (data not shown), we assumed that 75.7% of the absorbed photons were allocated to photoreactions in the PSII.

### 5. ^13^C Incubation

Twenty-two ^13^C incubation experiments were conducted (see Table 1 for dates and stations). A photosynthetron (modified from Babin et al., [8]) was used to perform *in situ* incubations. A U-shaped dimmable fluorescent tube (OSRAM, DULUX L, 2G11, 55W/12–950, LUMILUX DE LUXE, daylight) produced the light, and the temperature in the photosynthetron was maintained at the *in situ* temperature by a seawater circuit. Immediately after sampling, six litres of seawater were inoculated with NaH^13^CO_3_ (98 atom %, Sigma) corresponding to an enrichment of about 15% of the dissolved inorganic carbon already present in the seawater. The inoculated seawater was shared among 20 culture flasks (265 mL) placed in the photosynthetron. Light intensity was measured in each flask using a micro-spherical quantum sensor (US-SQS; Walz) connected to a LI-COR 1400 data logger, and one flask was maintained in the dark to estimate the non-photosynthetic inorganic carbon incorporation. After three hours of incubation, each flask was filtered onto 25 mm pre-combusted (450°C, 12 h) GF/F filters and stored at −20°C until analysis. To remove carbonates, filters were exposed to fuming HCl for four hours and then dried at 50°C for 12 hours. The particulate organic carbon (POC) concentration and the isotopic ratio of ^13^C to ^12^C were determined using an elemental analyzer (EA 3000, Eurovector) combined with a mass spectrophotometer (IsoPrime, Elementar). The carbon fixation rate (P_obs_) was calculated according to Hama et al. [34]. The value for incorporation in the dark was subtracted from all data and P_obs_ was expressed in µmol C L^−1^ h^−1^. Each P_obs_ vs. E curve was then performed on 20 values.

**Table 1:** R_max_, E_Rmax_ and γ values per date and station.

Date	Station	R_Max_	E_Rmax_	γ
13-Mar-10	4	85.54	349	0.152
13-Mar-10	5	63.17	0	−0.097
5-May-10	3	24.37	876	0.040
5-May-10	5	49.13	4143	0.055
5-May-10	7	10.74	422	0.036
2-Jun-10	3	26.87	394	0.079
2-Jun-10	6	15.31	590	0.040
2-Jun-10	9	4.70	405	0.013
7-Jul-10	4	6.99	368	0.019
7-Jul-10	7	2.54	210	0.013
7-Jul-10	9	5.91	205	0.030
20-Aug-10	4	11.61	143	0.005
20-Aug-10	7	5.49	14	−0.004
20-Aug-10	9	10.09	238	0.016
29-Sep-10	4	21.32	0	−0.024
29-Sep-10	7	7.14	222	0.028
29-Sep-10	9	5.80	328	0.012
22-Oct-10	4	10.38	0	−0.007
22-Oct-10	7	6.58	0	−0.002
22-Oct-10	9	8.66	297	0.018
7-Dec-10	4	10.01	83	−0.003
7-Dec-10	7	8.89	192	0.025

R_max_ is in mol e^−^ mol C^−1^, E_Rmax_ is in µmol photons m^−2^ s^−1^ and γ is in mol e^−^ mol C^−1^ (µmol photons m^−2^ s^−1^)^−1^.

### 6. P vs. E Curve

The ETR and P_obs_ were plotted against light (E). To estimate photosynthetic parameters, the mechanistic model of Eilers and Peeter [35] was applied to the data:
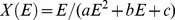
(8)where X (E) is ETR (E) or P_obs_ (E). The maximum photosynthetic capacity was calculated as follows:
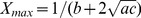
(9)where X_max_ is the maximum photosynthetic capacity measured with the PAM method (ETR_max_ in µmol electrons L−1 h−1) or with the ^13^C incubation method (P_max_ in µmol C L−1 h−1).

The ETR/P_obs_ ratio was plotted against light intensity. As a consequence of the use of the mechanistic model of Eilers and Peeter [35] to fit the data, we were able to estimate the relationship between ETR/P_obs_ and light for each curve by:
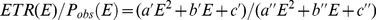
(10)Where a’, b’ and c’ are the estimated parameters of the mechanistic model of Eilers and Peeter [35] for the ETR vs. light relationship and a”, b” and c” for the P_obs_ vs. light relationship.

**Figure 2:**
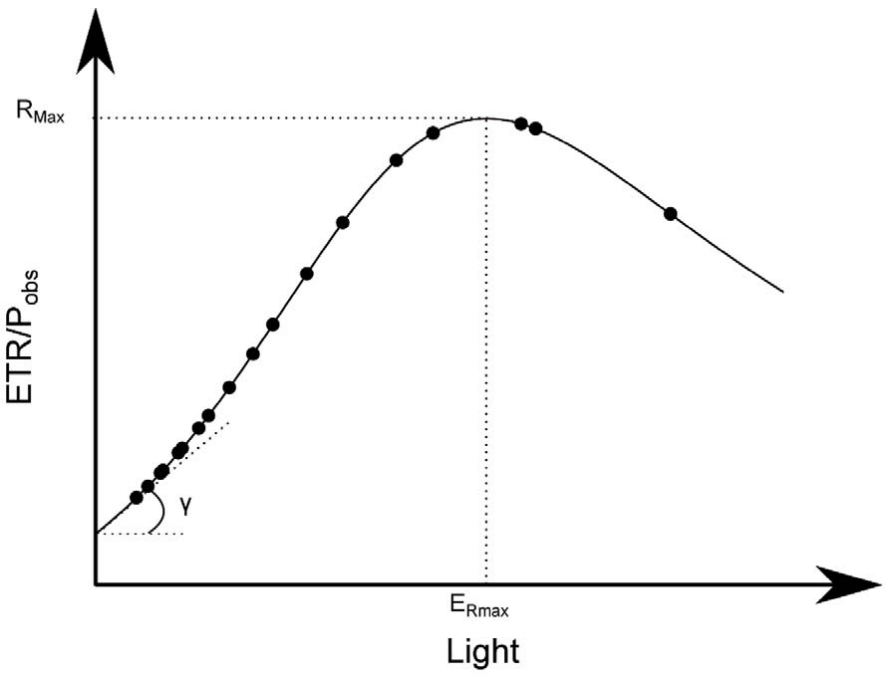
Example of relationship between ETR/P_obs_ ratio and light. R_max_ is the maximum value of ETR/P_obs_, E_Rmax_ is the light intensity at which R_max_ is measured and γ the initial slope of the curve.

Three parameters were defined to describe the relationship between ETR/P_obs_ and light intensity (Figure 2): R_max_, the maximum value of ETR/P_obs_ in mol e^−^ mol C^−1^; E_Rmax_, the light intensity at which R_max_ was measured in µmol photons m^−2^ s^−1^; γ, the initial slope of the curve in mol e^−^ mol C^−1^ (µmol photons m^−2^ s^−1^)^−1^. A Ward’s clustering was performed on the three parameters to identify data with the same shaped curve.

The relationships between the shapes of the ETR/P_obs_ vs. light and physicochemical and biological parameters were examined by principal component analysis (PCA) (software R 2.11.1).

### 7. Data Analyses

Logarithmic regression analysis was carried out on the full dataset to study the relationship between P_obs_ and ETR using SigmaPlot 11.0 (Systat Software). The logarithmic relationship obtained gave us a model, *Sim_ETR_1*, which enabled us to estimate the rate of carbon fixation (P_simETR1_) using ETR data.

To improve the first *Sim_ETR_1* model, the differences between P_obs_ and P_simETR1_ were calculated. Linear dependences between physicochemical and biological parameters and P_obs_-P_simETR1_ (ΔP) were carried out using the Pearson product moment correlation of SigmaPlot 11.0 (Systat Software) to identify the parameters that influenced the relationship between the rate of carbon fixation and ETR. After electing the parameters using the method of forward stepwise regression, a multiple linear regression was performed to estimate ΔP (SigmaPlot 11.0). This estimation was added to *Sim_ETR_1* to form *Sim_ETR_2*.

Linear regression analyses were performed between P_obs_ and models (*Sim_ETR_1* and *Sim_ETR_2*) to estimate the significance of these relationships.

## Results

### 1. Carbon Incorporation Estimation Versus ETR

**Figure 3:**
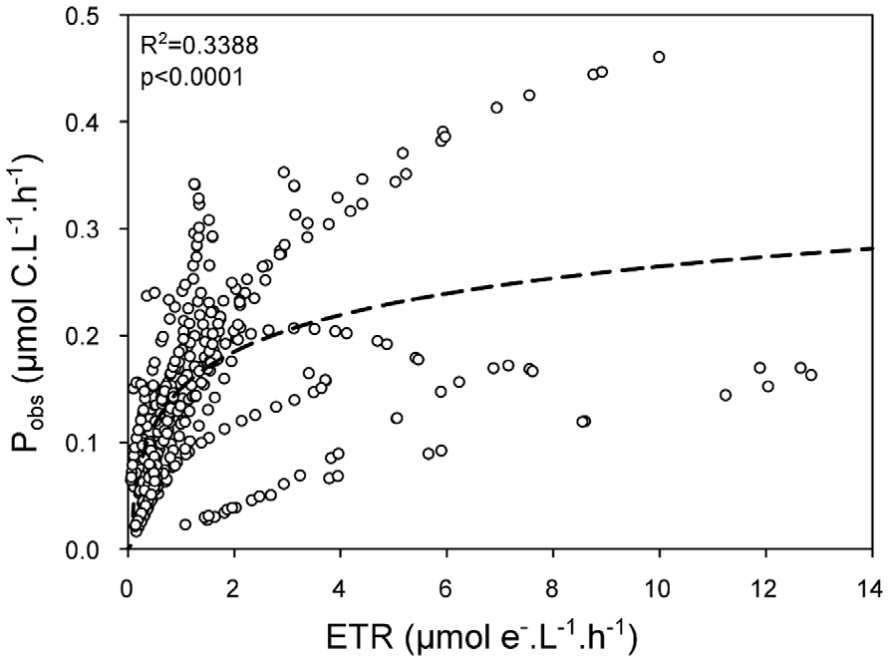
Observed ^13^C incorporation (P_obs_) plotted against the electron transport rate (ETR). The dotted line represents the logarithm regression (P_obs_  = 0.1503+0.0496 * ln(ETR)).

To investigate the relationship between the carbon incorporation and the ETR measurements for our data set measured in the English Channel, we plotted the carbon incorporation observation (P_obs_), estimated using ^13^C, against ETR (Figure 3). A significant linear relationship was found between P_obs_ and ETR up to 2 µmol e^−^ L^−1^ h^−1^ (R^2^ = 0.4022, P_obs_  = 0.138 * ETR, p<0.001). After this point, the relationship was no longer linear and P_obs_ values tended to level off at high ETR values. Consequently, this relationship can be described by a logarithmic function and the dataset was therefore fitted to a two-parameter logarithm curve (Figure 3). Using the logarithm curve, a significant fit (p<0.0001, P_obs_  = 0.1503+0.0496 * ln(ETR), equation 11) was obtained with a relatively high R^2^ value (0.3388).

**Figure 4:**
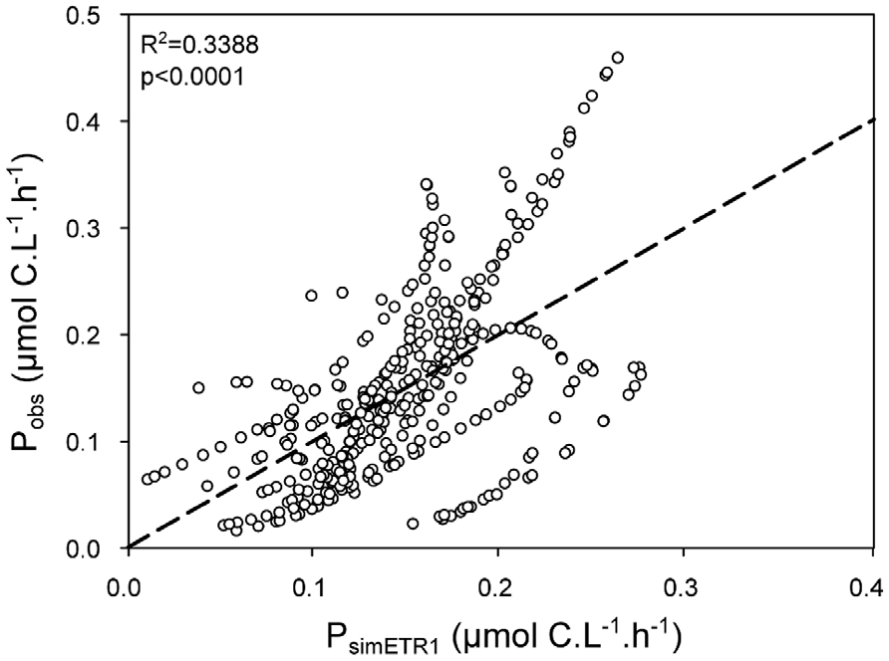
Observed ^13^C incorporation (P_obs_) plotted against estimated carbon incorporation by the equation *Sim_ETR_1*. P_simETR1_ = 0.1503+0.0496 * ln(ETR). The dotted line represents the linear regression (P_obs_  = 1 * P_simETR1_).

With a view to estimating PP by ETR, the rate of carbon incorporation was calculated by transforming ETR data with equation 11 determined by the logarithm regression. P_obs_ and our first estimation of carbon incorporation (*Sim_ETR_1,* P_simETR1_ = 0.1503+0.0496 * ln(ETR), equivalent to equation 11), was in relatively good agreement (R^2^ = 0.338, p<0.0001) (Figure 4).

To highlight the physicochemical and biological parameters which drive the relationship between PP and ETR, and to improve the estimation of PP by using ETR, the correlation coefficients of the difference between P_obs_ and P_simETR1_ (ΔP) and physicochemical and biological parameters were calculated (Table 2). High negative correlation coefficients were obtained between ΔP and nutrient concentrations (r_ΔPvDIP_ = −0.701, r_ΔPvDIN_ = −0.488, r_ΔPvDSi_ = −0.422). ΔP was also highly negatively correlated with F_v_/F_m_ (r_ΔPvFv/Fm_ = −0.475) and positively with irradiance (r_ΔPvPAR_ = 0.386). However there were also low correlations between ΔP and the Chl*a* concentration (r_ΔPvChl*a*_ = 0.250) and the SPM concentration (r_ΔPvSPM_ = 0.104) and no correlation with the dinoflagellate:diatom ratio (r_ΔPvDino/Diat_ = 0.046).

**Table 2:** Linear correlation coefficients (Pearson) between ΔP and physicochemical and biological parameters.

Parameters	PAR	Temp	DIP	DIN	DSi	Chl*a*	SPM	F_v_/F_m_	Dino/Diat
**ΔP**	0.386	0.351	−0.701	−0.488	−0.422	0.250	0.104	−0.475	0.046

PAR: incident light in µmol photons m^−2^ s^−1^, Temp: temperature in °C, DIP concentrations in µmol L^−1^, DIN concentrations in µmol L^−1^, DSi concentrations in µmol L^−1^, Chl*a* biomass in µg L^−1^, SPM concentrations in mg L^−1^ and F_v_/F_m_, and dinoflagellate:diatom ratio.

Parameters used to improve the estimation of PP by ETR were selected using forward stepwise regression. According to this method, the variables DIP and PAR were selected. Due to the high correlation coefficients between DIP, DIN and DSi concentrations [23] only the variable DIP was used in the model, even though high correlation between ΔP and all nutrient concentrations were found.

**Figure 5:**
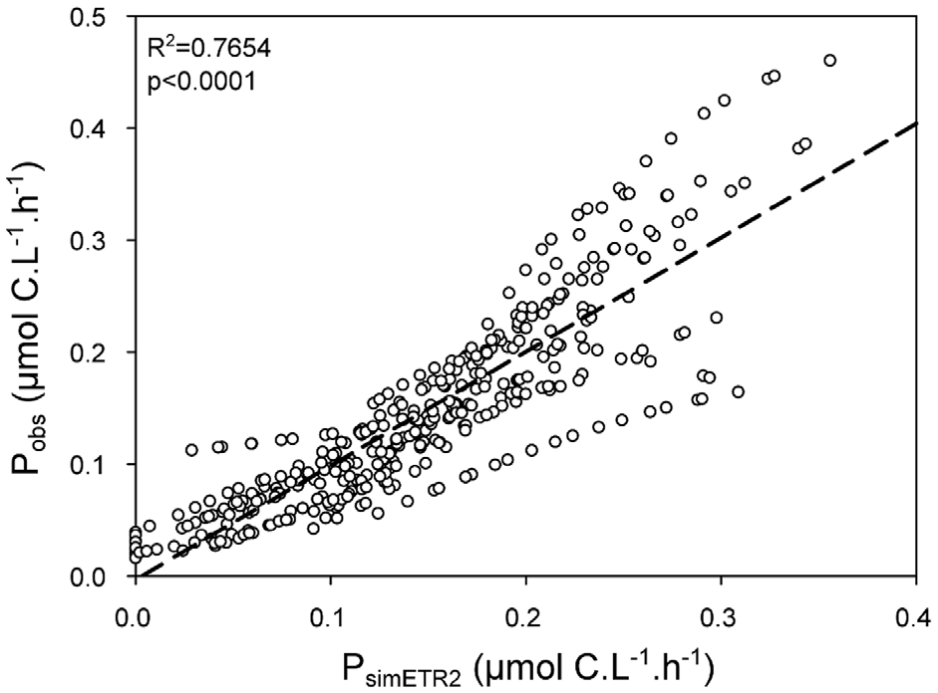
Observed ^13^C incorporation (P_obs_) plotted against estimated carbon incorporation by the equation *Sim_ETR_2*. P_simETR2_ = 0.2082+0.0496 * ln(ETR) - (0.319 * DIP) + (0.000166 * PAR). The dotted line represents the linear regression (P_obs_ = −0.0031+1.0175 * P_simETR2_).

A multiple linear regression was performed to estimate ΔP as a function of PAR and DIP. The result of the multiple linear regression was added to *Sim_ETR_1* to improve the carbon incorporation estimation by ETR. The second estimation of carbon incorporation (*Sim_ETR_2,* P_simETR2_ = 0.2082+0.0496 * ln(ETR) - (0.319 * DIP) + (0.000166 * PAR), equation 12) based on multiple linear regression approach was in good agreement with P_obs_ and the value of the R^2^ was high, 0.7654 (p<0.0001) (Figure 5).

### 2. Understanding the Relationship between Carbon Incorporation and ETR

The ratio between ETR and P_obs_ was plotted against light intensity. Each curve was fitted using equation 10. To better understand this relationship, three parameters were defined. These parameters allowed the curve of the ETR/P_obs_ vs. the light relationship to be described. R_Max_ represents the maximum value of ETR/P_obs_ estimated and E_Rmax_ the light intensity at which R_Max_ was measured. The parameter γ is the initial slope of the curve (Figure 2). The three parameters varied markedly over the data set (Table 1). R_Max_ varied from 2.54 to 85.54 mol e^−^ mol C^−1^, E_Rmax_ varied from 0 to 4143 µmol photons m^−2^ s^−1^ and γ varied from −0.097 to 0.152 mol e^−^ mol C^−1^ (µmol photons m^−2^ s^−1^) ^−1^.

**Figure 6:**
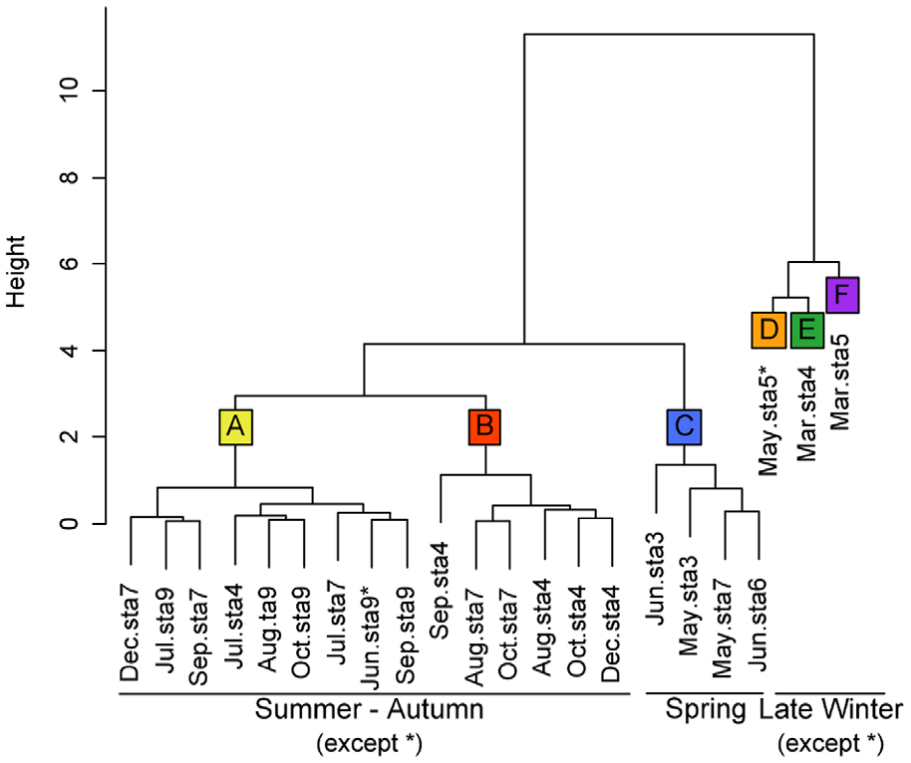
Tree topology obtained by Ward’s clustering performed on R_max_, E_Rmax_ and γ.

**Figure 7:**
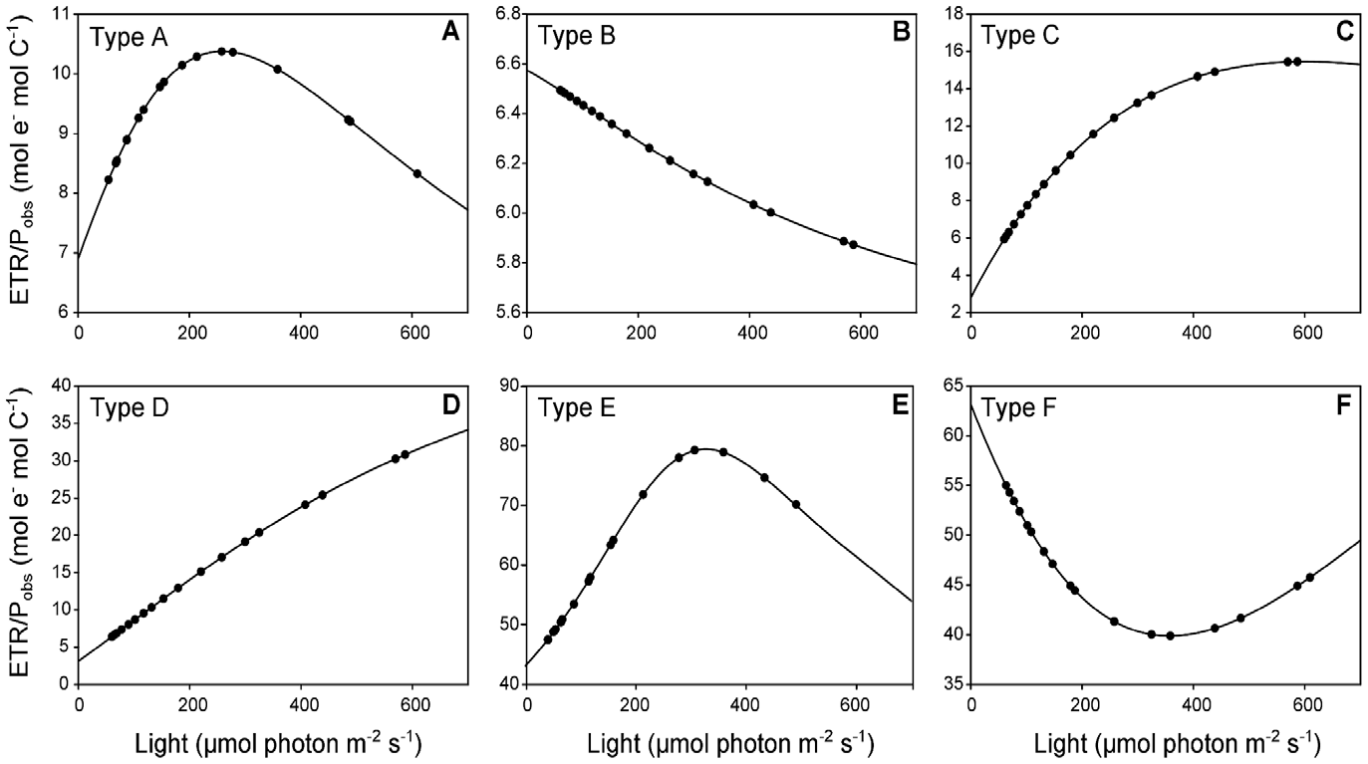
Relationships between the ETR/P_obs_ ratio and light showing the different shaped curves obtained. (A) in August 2010 at station 9 corresponding to a Type A shape, (B) in October 2010 at station 7 corresponding to a Type B, (C) in June 2010 at station 6 corresponding to a Type C, (D) in May 2010 at station 5 corresponding to a Type D, (E) in March 2010 at station 4 corresponding to a Type E and (F) in March 2010 at station 5 corresponding to a Type F.

The typology of Ward’s clustering performed on R_max_, E_Rmax_ and γ enabled the data to be divided into six groups (A, B, C, D, E and F) (Figure 6). Each group was characterized by the shape of the curve describing the relationships between the ETR/P_obs_ ratio and the light. Examples of the different shapes are shown in Figure 7.

**Figure 8:**
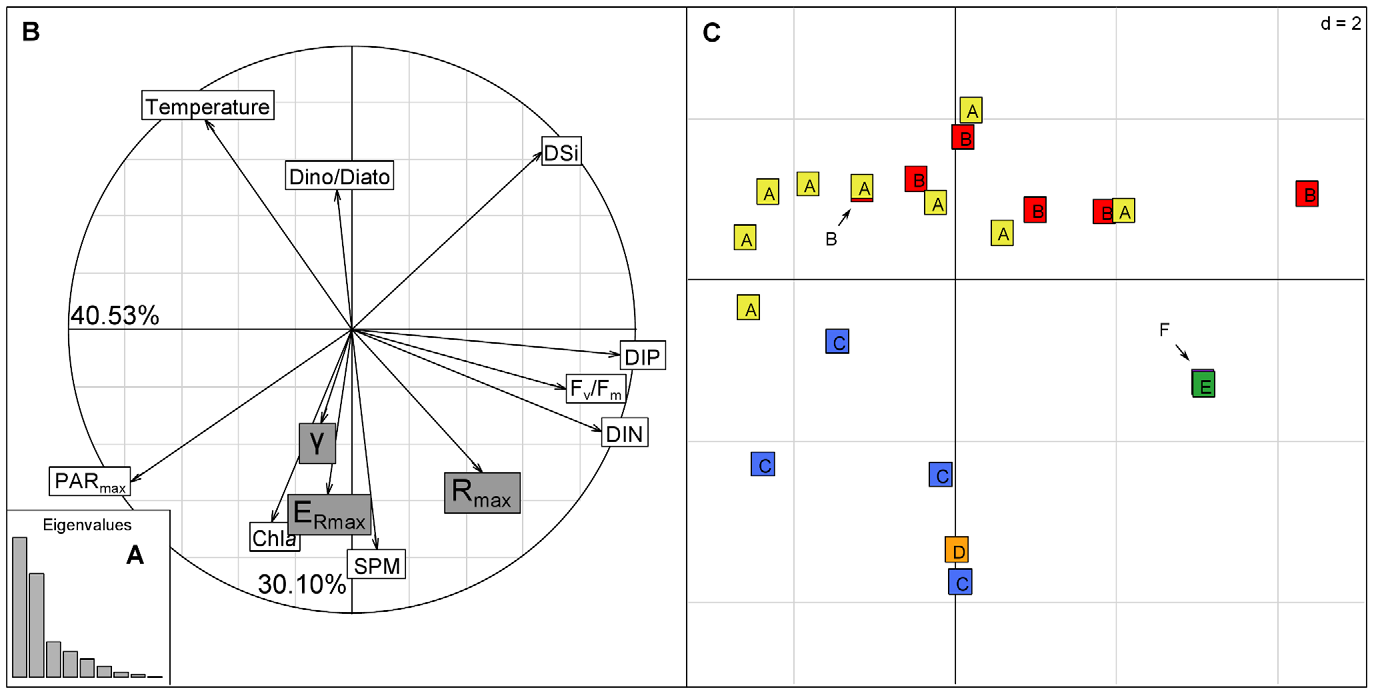
Principal Component Analysis highlighted the link between physicochemical and biological parameters and the 6 different curve shapes. (A) Histogram of eigenvalues, (B) Principal Component Analysis (PCA) plot of factor loadings in the plane defined by the two first axes. White labels refer to active variables and grey labels to illustrative variables, (C) Sample ordination plot. A, B, C, D, E and F refer to groups defined by Ward’s clustering.

PCA was used to highlight the link between physicochemical and biological parameters and the six curve shapes defined by Ward’s clustering. The two first axes of the PCA explained more than 40% and 30% of the total inertia, respectively (Figure 8A). Therefore the analysis of the PCA was based on the two first axes. DIN, DIP and DSi were related to the first component whereas the biological parameters Chl*a* biomass, dinoflagellate:diatom ratio and SPM were related to the second component (Figure 8B). According to the factor loadings plot of the PCA and to the coefficients of correlation (data not shown), R_max_ was positively correlated with DIP, DIN and F_v_/F_m_ and negatively with temperature, whereas E_Rmax_ and γ were positively correlated with the Chl*a* biomass and the SPM was negatively correlated with the dinoflagellate:diatom ratio and DSi.

The sample ordination plot (Figure 8C) shows that the shape of the ETR/P_obs_ vs. light curves depended on the physicochemical and biological parameters. The six curve shapes were associated with specific environmental conditions. A high R_max_ characterised groups E and F which were associated with high nutrient concentrations. High γ and E_Rmax_ characterised groups C and D, which were positively correlated with Chl*a* biomass, SPM and PAR_max_ and negatively correlated with the dinoflagellate:diatom ratio and the DSi concentration. Low γ and E_Rmax_ generally characterized groups A and B associated with low Chl*a* concentrations and a high dinoflagellate:diatom ratio. Moreover, A and B were associated with the first axis mainly built by the nutrient concentrations, revealing the high influence of nutrients.

### 3. Estimation of the Primary Production Capacity using ETR

**Figure 9:**
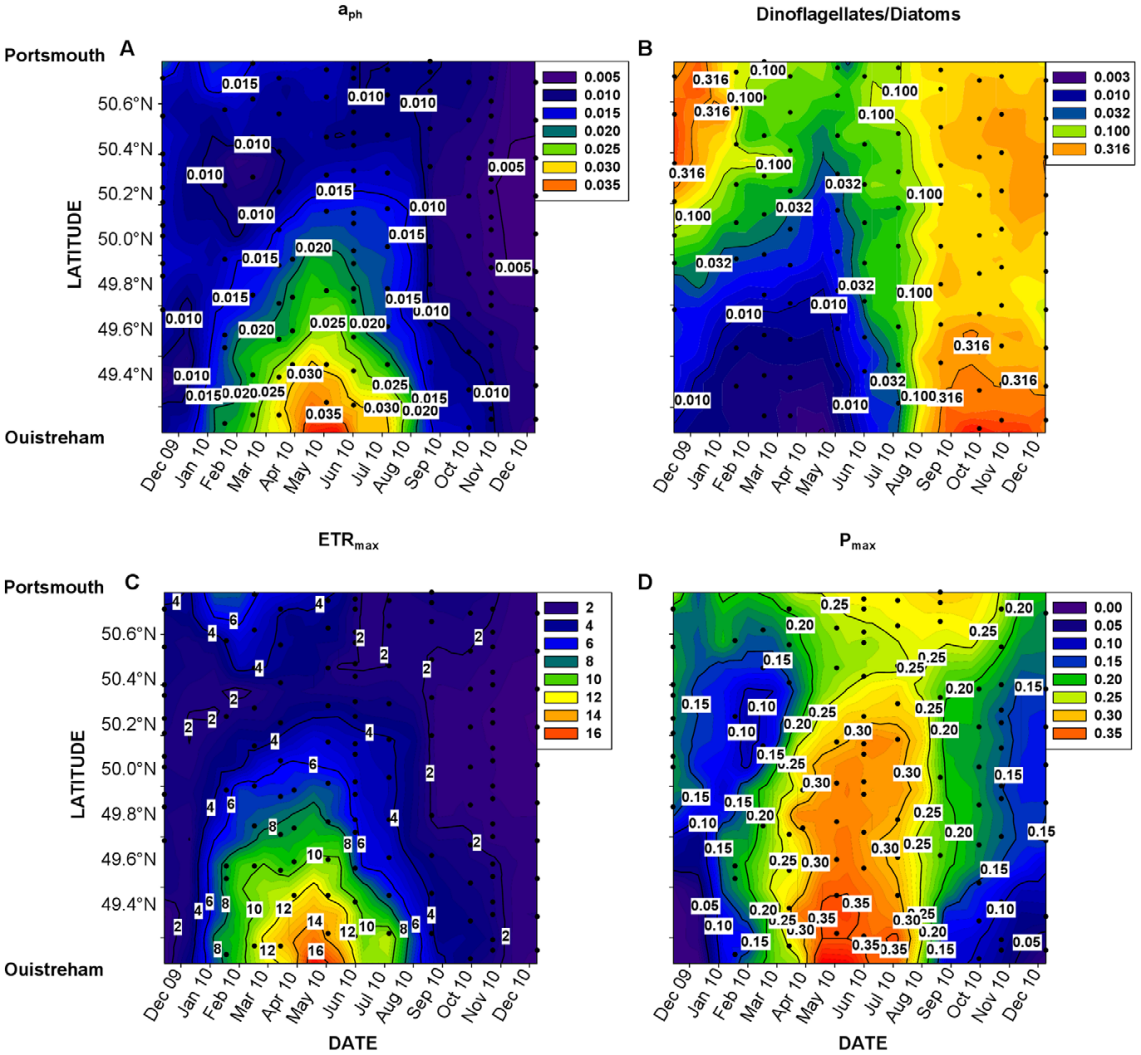
Latitude-time distributions of a_ph_, the dinoflagellate:diatom ratio, ETR_max_ and P_max_. (A) a_ph_ in m^−1^, (B) the dinoflagellate:diatom ratio (C) ETR_max_ the maximum photosynthetic capacity (maximal electron transport rate – ETR_max_) in µmol e^−^ L^−1^ h^−1^ and (D) P_max_ maximum estimated carbon incorporation using the *Sim_ETR_2* model in µmol C L^−1^ h^−1^.

Carbon incorporation (P_obs_) was measured at a low frequency (22 measurements) whereas the other parameters including PAM were measured at a high frequency (358 measurements) from November 2009 to December 2010. The paper by Napoléon et al. [23] presented the complete dataset of rETR (relative values) and the results of monitoring the dynamics of rETR throughout the year. In order to estimate PP, the *Sim_ETR_2* model described above was applied to the complete dataset from the central English Channel after the transformation of rETR data into ETR, using a_ph_ data measured during those cruises (Figure 9A). The spatiotemporal variability of a_ph_ revealed a clear seasonal pattern with higher values in winter/spring i.e. between March and July from the French coasts to the centre of the English Channel. The highest value of 5.27 10^−2^ m^−1^ was recorded in March at latitude 49.37. The lowest value of 3.46 10^−4^ m^−1^ was measured in February at latitude 50.16.

The dinoflagellate:diatom ratio remained low throughout the year of study (Figure 9B) and was related to a_ph_. The lowest values were recorded between November 2009 and June 2010 from the French coast to the centre of the English Channel. The highest value of 11.98 was recorded in September near the French coast.

ETR_max_ was highly variable over time and space (Figure 9C). A peak was observed between March and June on the French coast with a maximum value of 24.12 µmol e^−^ L^−1^ h^−1^. The minimum value of 0.34 µmol e^−^ L^−1^ h^−1^ was recorded in May at latitude 50.47.

The result of the conversion of the ETR_max_ data into an estimated maximum carbon incorporation (PP_max_) using the *Sim_ETR_2* model is shown in Figure 9D. From March to June, PP_max_ values higher than 0.37 µmol C L^−1^ h^−1^ were calculated from the French coast to latitude 49.71. A smaller summer peak was observed from the English coast to latitude 50.65 with PP_max_ values higher than 0.25 µmol C L^−1^ h^−1^. The lowest values were measured in winter.

## Discussion

### 1. Carbon Incorporation Measurements vs. ETR

The ^13^C method was used to estimate carbon incorporation (P_obs_). P_obs_ measurements were then plotted against ETR measurements. A logarithmic relationship was found between P_obs_ and ETR, with an initial slope of 0.138 mol C (mol e^−^)^−1^ which is in agreement with values reported in the literature [12], [17]. Similar non-linear relationships between oxygen production or carbon incorporation and electron transport rates were also found in planktonic microalgae by Geel et al. [36], Flameling and Krompkamp [19], Masojidek et al. [37] and Schaeffer et al. [38].

Alternative electron sinks at high irradiance may explain such a relationship to protect the phytoplankton from photoinhibition by damage to the PSII under high irradiance. Indeed, electrons produced from the PSII are involved in the production of reducing equivalents and ATP subsequently required for carbon fixation. However, besides this linear transport of photosynthetic electrons, there are other ways for electrons cycling and divergence of electrons occurs with other reactions [39]. Several physiological mechanisms may explain these electron sinks: photorespiration involving the oxygenase activity of the Rubisco; the Melher reaction, which involves O_2_ as the last acceptor of electrons in the PSI instead of NADP^+^; nitrate reduction by nitrate reductase, which can also be an electron sink [39].

Photorespiration probably plays a minor role as shown by Geel et al. [36], Flameling and Krompkamp [19] and Claquin and al. [18]. The apparent absence of photorespiration has often been described in algae [40] and particularly in diatoms, which have an efficient CO_2_-concentration mechanism [18], [41], [42].

Higher Mehler activity at high irradiance can be another electron sink rather than photorespiration according to Geel et al. [36], Flameling and Kromkamp [19] and Claquin et al. [18]. This pseudocyclic electron transport can represent between 50% to 60% of the oxygen uptake stimulated by light at medium and high irradiances [18].

The reduction of nitrate can lead to a significant loss of photosynthetic electrons [43]. In diatoms, nitrate uptake and reduction occur even in the absence of metabolic demand. Under no nitrogen limitation, diatoms, which represented the largest biomass in our samples, are able to transform excess photosynthetic energy into nitrate reduction [43].

In addition, an increase in dark respiration (mitochondrial respiration) may also explain the difference between the rate of carbon fixation and ETR [19]. Indeed, mitochondrial respiration is higher in the light than in the dark [18], [44], [45]. Mitochondrial activity is probably limited by the concentration of substrate in the dark. In the light, the production of photosynthates probably increases mitochondrial respiration by providing more substrate for the Calvin cycle [46]. This process could thus play a role in the non-linear relationships observed between P_obs_ and ETR at high light intensities. The experiments conducted in the present study did not make it possible to define the proportion of each sink in the deviation between P_obs_ and ETR.

The relationship between P_obs_ and ETR appeared to vary as a function of physicochemical and biological parameters. The latitude-time distribution of these parameters is detailed in Napoléon et al. [23]. Nutrient concentrations and the quantum efficiency of the PSII (F_v_/F_m_), often used as an indicator of nutrient stress [11], were negatively correlated with ΔP, indicating overestimation of *Sim_ETR_1* in non limiting nutrient conditions. The absence of nutrient limitations leads to high production of photosynthetic energy and to an imbalance between the generation of energy and the energy needed for growth. The electrons sinks described above, which play a protective role, may explain this regulation. In this case, the role of the nitrate reductase can be put forward. Over the year of study, DIN varied from 0.12 to 167 µmol L^−1^ [23]. Under high DIN concentrations, the uptake in excess of nitrate and its reduction by the nitrate reductase could have played a major role in energy overflow metabolism [12], [43]. As a consequence, reduced nitrate can be released to the surrounding environment [43]. The effect of phosphate concentration, which varied from 0.06 to 1.75 µmol L^−1^ [23], appeared to play a role but the mechanisms were not clear. Thus, the relation between carbon incorporation and ETR cannot be described by a simple relation but certainly involves many factors, especially light and nutrient variables, in modulating the relationship between P_obs_ and ETR because of the deviation at high light intensity and as a function of nutrient concentrations. The effect of nutrient limitations probably varies with the nutrient concerned [43], [47] but in the present work, the strong correlation observed between all nutrients made it impossible to determine the specific effect of each individual nutrient.

According to the low correlation between ΔP and the dinoflagellate:diatom ratio, the relationship between P_obs_ and ETR did not depend on the structure of the community. However, a_ph_, used to transform rETR data into ETR data, varied considerably with species composition [29]. The effect of the species composition was consequently taken into account in the calculation of ETR.

However, caution is required here, because a high correlation does not necessary imply a causal effect. The parameters taken into account could be good integrators of seasonality, without acting directly on the ETR vs. P_obs_ relationship.

### 2. Understanding the Relationship between Carbon Incorporation and ETR

The light intensity drives the relation between carbon incorporation and ETR as described above. Some authors have taken the slope coefficient and the intercept of the linear regression of the P_obs_ vs. ETR relationship into account [12], [17] but none has accounted for changes in the ETR/P_obs_ ratio as a function of light. In our study, to better understand this relationship, three parameters were defined to describe changes in the ETR/P_obs_ ratio as a function of the light intensity. E_Rmax_ indicates the light intensity at which the ratio between ETR and P_obs_ reached maximum (R_max_). γ, the initial slope expresses the efficiency of carbon fixation in moles of carbon per mole of electrons produced as a function of the light. Thus, six curve shapes were described for the whole dataset which were in accordance with the seasonal cycle e.g. shapes A and B correspond to summer and autumn data, shapes C and D correspond to spring, and shapes E and F to winter. In Barranguet and Kromkamp [12], the absence of seasonality of the conversion factor of ETR to PP was observed for microphytobenthos. On the contrary, our results highlight the importance of the seasonal cycle of the physicochemical parameters and/or biological parameters in the relationships between the ETR/P_obs_ ratio and light intensity. This can be explained by the different drivers encountered in our system versus in benthic systems.

The relationship between the ETR/P_obs_ ratio vs. light showed that the losses of electrons in other ways than the fixation of carbon increased with light intensity up to the E_Rmax_ intensity as described above. High E_Rmax_ were recorded in spring (types C and F) and low E_Rmax_ in summer and autumn (types A and B). Seasonal changes in the structure of the community may explain such variations. Glee and al. [36] showed that the light intensity at which the deviation between carbon incorporation and ETR takes place depends on the algal species and can vary from 200 to 1000 µmol photon m^−2^ s^−1^. In our study, low dinoflagellate:diatom ratios were recorded from the end of winter to the end of spring while high values were recorded from the end of spring to the end of autumn due to an increase in the dinoflagellate biomass. Beyond the E_Rmax_ intensity, the slight decrease in the ETR/P_obs_ ratio means that the production of electrons in the PSII was affected at high light intensity, whereas dark reactions of photosynthesis were not damaged.

Low values of the parameter γ were recorded in summer and autumn (types A and B) and high values in spring (types C). Low values reveal the incapacity of the cells to respond to an increase in light and early photoinhibition, while high values show that phytoplankton cells are able to cope with the increase in electron production with light without damaging the PSII. E_Rmax_ and γ covaried because they both characterise the capacity of the phytoplankton to respond to changing light and to adapt to low or high light intensity. E_Rmax_ and γ were positively correlated with PAR_max_, revealing a high capacity of the cells growing under high light intensity to respond to the increase in light. At high light intensity the alternative electron sinks offset the high rate of electron production. Conversely, low values of E_Rmax_ and γ reveal that high photoinhibition mechanisms came into play with an increase in light. It thus appears that photoinhibition mechanisms can occur at lower light intensities in the case of phytoplankton adapted to low light. The efflorescence of dinoflagellates was correlated with low values of E_Rmax_ and γ, revealing that dinoflagellate cells were less able to respond to changing light conditions than diatom cells [48].

R_max_ represents the highest ETR/P_obs_ preceding the occurrence of photoinhibition mechanisms. High values of R_max_ recorded in types E and F were correlated with high nutrient concentrations and high F_v_/F_m_. We thus suggest that high R_max_ values reflect the high capacity of a cell to cope with an unbalance between energy production and the energy it requires for growth. Nutrient limitations could play an important role in determining cell susceptibility to photoinhibition [49]. In particular, photoinhibition is higher for cells limited by nitrogen [50]. In fact, the capacity to respond to changing light is affected by nutrient stress [20], [51]. Furthermore, R_max_ was negatively correlated with temperature. These results are consistent with those of Morris and Krompkamp [17]. The slope coefficient of the ETR vs. P_obs_ relationship was negatively affected by temperature from 5°C to 20°C, which was the range of temperature recorded in our area over the year of study [23].

### 3. Estimation of the Primary Production Capacity in the Central English Channel

By using this approach and the *Sim_ETR_2* model, we were able to estimate the maximum primary production (PP_max_) in the central part of the English Channel between November 2009 and December 2010. Low frequency data of carbon incorporation measurements (P_obs_) were combined with the high frequency data of PAM measurements detailed in Napoléon et al. [23].

The a_ph_ values allowed us to transform the rETR_max_ dataset (relative unit) into ETR_max_ data. The range of a_ph_ is in accordance with those cited in the literature [30]. A seasonal variability of a_ph_ was revealed with higher values in winter/spring. The seasonality of a_ph_ depends to a great extent on the dynamics of the phytoplankton biomass [23] and is also due to the package effect [30], [52] which varies as a function of photoacclimation and with the phytoplankton species. Johnsen and Sakshaug [29] showed that the values of the specific absorption cross-section (a*) (data not shown) were higher for dinoflagellates than diatoms, which is in agreement with the high a* values we measured during summer when the dinoflagellate:diatom ratio was high.

The levels of productivity (data not shown) estimated in the present study using the *Sim_ETR_2* model reached values of 10 mgC mg Chl*a* ^−1^ h^−1^ at the beginning of summer which is in agreement with measurements made by Jouenne et al. [53], Pannard et al. [3] and Claquin et al. [51] with maximum values of 8.88, 8.48 and 17 mgC mg Chl*a* ^−1^ h^−1^ respectively. The dynamics was also in agreement with the results of Jouenne et al. [53] in a French estuarine bay of the English Channel (Veys Bay). The French coastal area had a higher production rate than the English coastal area and the highest rate of production was measured from April to July whereas in the English coastal area, the rate of production was low but constant from April to the end of October. As described in Napoléon et al. [23], the French coastal area receives high freshwater inputs whereas low nutrient concentrations were recorded in the English coastal area, and this is the main explanation for the difference in dynamics between these two coastal areas (see Napoléon et al. [23] for details).

### Conclusion

We have highlighted the non-linear relationship between ETR measurements and P_obs_ and underlined the importance of taking physicochemical parameters like the incident light or nutrient concentrations into account to understand and estimate PP using ETR data. By combining the two methods, we have shown that it is possible to access the high spatial and temporal dynamics of primary production.

However, we used an empirical model. To confirm the hypotheses we put forward to explain the ETR vs. P_obs_ relationship, experiments under controlled conditions are required.

The same type of approach should be used for other ecosystems to explore in more detail the parameters which control the relation between PP and ETR. In addition, extensive experiments on monoclonal cultures belonging to different phyla under different conditions are necessary to hierarchize the parameters controlling this relationship.
